# Clinical Presentation and Outcomes Following Infection With *Vibrio* spp, *Aeromonas* spp, *Chromobacterium violaceum*, and *Shewanella* spp Water-Associated Organisms in Tropical Australia, 2015–2022

**DOI:** 10.1093/ofid/ofae319

**Published:** 2024-06-12

**Authors:** Stuart Campbell, Kirsten MacGregor, Emma L Smith, Tanmay Kanitkar, Sonja Janson, Robert W Baird, Bart J Currie, Sudharsan Venkatesan

**Affiliations:** Departments of Medicine and Pathology, Royal Darwin Hospital, 105 Rocklands Dr, Tiwi, Darwin 0810, Australia; Departments of Medicine and Pathology, Royal Darwin Hospital, 105 Rocklands Dr, Tiwi, Darwin 0810, Australia; Department of Infection Sciences, North Bristol NHS Trust, Bristol, UK; Departments of Medicine and Pathology, Royal Darwin Hospital, 105 Rocklands Dr, Tiwi, Darwin 0810, Australia; Menzies School of Health Research, Charles Darwin University, Darwin, Australia; Departments of Medicine and Pathology, Royal Darwin Hospital, 105 Rocklands Dr, Tiwi, Darwin 0810, Australia; Department of Infection, Kings College Hospital, London, UK; Departments of Medicine and Pathology, Royal Darwin Hospital, 105 Rocklands Dr, Tiwi, Darwin 0810, Australia; Departments of Medicine and Pathology, Royal Darwin Hospital, 105 Rocklands Dr, Tiwi, Darwin 0810, Australia; Departments of Medicine and Pathology, Royal Darwin Hospital, 105 Rocklands Dr, Tiwi, Darwin 0810, Australia; Menzies School of Health Research, Charles Darwin University, Darwin, Australia; Departments of Medicine and Pathology, Royal Darwin Hospital, 105 Rocklands Dr, Tiwi, Darwin 0810, Australia

**Keywords:** *Aeromonas*, *Chromobacterium*, *Shewanella*, *Vibrio*, water-associated infections

## Abstract

**Background:**

Water-associated bacterial infections cause a wide spectrum of disease. Although many of these infections are typically due to human host commensal *Staphylococcal* or *Streptococcal* spp, water exposure can result in infections with environmental gram negatives such as *Vibrio* spp, *Aeromonas* spp, *Chromobacterium violaceum*, and *Shewanella* spp (collectively VACS).

**Methods:**

We performed a retrospective analysis of the epidemiology, clinical presentation, and outcomes of deep and superficial infections associated with VACS organisms in our health service between 1 January 2015 and 31 December 2023.

**Results:**

We identified 317 patient episodes of infection with VACS organisms over this period. Of these, *Aeromonas* spp (63%) was the most common, followed by *Vibrio* spp (19%), *Shewanella* spp (13%), and *C violaceum* (5%). The majority were isolated from males (74.4%) and involved the lower limb (67.5%). Mild infections were more common than severe presentations, with only 15 (4.7%) admissions to the intensive care unit and 8 (2.5%) deaths. Colonization occurred in 6.9% of patients, in contrast to the perceived severity of some of these bacteria. Copathogens were common and included *Staphylococcus aureus* (48%) and enteric bacteria (57%). The majority of patients (60%) had no documented water exposure. Initial empiric antimicrobial therapy presumptively covered the susceptibilities of the isolated organisms in 47.3% of patients; however, a lack of VACS-covering empirical therapy was not associated with readmission.

**Conclusions:**

The isolation of a VACS organism in our setting was often not associated with documented water exposure, which has implications for empiric antimicrobial therapy. Severe disease and death were uncommon.

## Teaser

Water exposure is a risk for atypical infections, and we assessed clinical outcomes associated with a specific subgroup: VACS (*Vibrio* spp, *Aeromonas* spp, *Chromobacterium violaceum*, and *Shewanella* spp). These infections occur without water exposure and are typically superficial infections, though deaths still occur. This has implications for empirical therapy.

There is a large spectrum of illnesses attributable to water-associated bacterial infections. These range from uncomplicated cellulitis to diabetic foot infections, bone and joint infections, rapidly progressive necrotizing fasciitis (NF), and bacteremia. The severity of clinical manifestations depends on the organism and associated virulence factors, inoculum size, mechanism of entry, and patient risk factors.

Although these syndromes, even in the setting of water exposure, are often due to host commensal *Streptococcal* spp or *Staphylococcus aureus* [1], water exposure can increase the risk of infection with environmental gram-negative organisms, including *Vibrio* spp, *Aeromonas* spp, *Chromobacterium violaceum*, and *Shewanella* spp (VACS). These organisms can be found in fresh or saltwater environments and may be associated with contaminants (eg, mud or sewage) or, in some settings, drinking water [2]. Importantly, Australian national non–water-associated empirical antimicrobial guidelines for skin and soft tissue infection (SSTI) do not recommend therapy that will cover these organisms [3].

Pathogenic *Vibrio* spp are halotolerant and therefore mainly associated with saltwater exposures. Clinical manifestations range from simple SSTI to severe necrotizing syndromes, which are associated with comorbidities such as chronic liver disease, immunocompromised states, iron storage disorders, chronic kidney disease, and diabetes mellitus [4–6]. *Aeromonas* spp are typically associated with purulent wound infections or abscesses and are most common in previously healthy individuals with traumatic wounds exposed to freshwater [7]. Infection with *C violaceum* is rare but has been associated with abscess formation and high mortality rates, but findings linking comorbidities and mortality are inconsistent [8–10]. *Shewanella* spp infections are uncommon and most often described in relation to chronic wound infections in patients with diabetes. Other associated risk factors for infection include underlying liver disease, malignancy, chronic kidney disease, and immunosuppression [11, 12].

Previous work at our institution in tropical northern Australia investigated VACS pathogens from 468 clinical isolates (80% SSTI) over a 13-year period and identified *Aeromonas* spp as the most common (67%), followed by *Vibrio* spp (15%), *Shewanella* spp (13%), and *C violaceum* (5%), though clinical outcomes were not studied [13].

There is limited research characterizing the clinical presentations and outcomes of these infections. Further information can assist clinicians treating infections associated with VACS organisms and may help inform empirical antimicrobial guidelines where water exposure occurs. This study aimed to describe the epidemiology, clinical presentation, and clinical outcomes for deep and superficial infections associated with VACS organisms in tropical northern Australia.

## METHODS

We conducted a retrospective cohort study to describe the clinical presentation, epidemiology, and outcomes for patients who presented with SSTI, osteoarticular, or bacteremic infections associated with VACS organisms to the Top End Health Service from 1 January 2015 through 31 December 2022. As we sought to investigate infections related to environmental exposure, patients with VACS isolations from other sites (stool, sputum, urine) were excluded.

The Top End Health Service comprises 3 hospitals: the Royal Darwin and Palmerston hospitals (RDPH) in the coastal capital of Darwin, Katherine District Hospital (KDH) in the subtropical interior, and Gove District Hospital (GDH) in the deep-water port of Nhulunbuy, which are spread across ∼500,000 km^2^ (Supplementary Figure 1). The climate is dominated by the summer monsoon, with interior freshwater floodplains emptying into the Timor Sea or Gulf of Carpentaria over the course of the relatively arid winter. This environment creates many opportunities for occupational or recreational water exposure and subsequently the risk of water-associated infections [4, 14].

Cases were identified through the Top End Health Service laboratory system (Labtrak; Intersystems) by searching for all VACS isolates in the study period. Repeat isolations ≤90 days from the index episode were excluded.

Isolates were identified by biochemical and phenotypic methods, including API 20NE (bioMérieux), Vitek 2 (bioMérieux), and/or matrix-assisted laser desorption/ionization time-of-flight mass spectroscopy (bioMérieux). Cases with ≥1 VACS organism isolated were aggregated into the more pathogenic species, which we graded in order of decreasing virulence from *C violaceum* to *Vibrio* spp, *Shewanella* spp, and finally *Aeromonas* spp.

Anonymized data were recorded with the REDCap electronic data capture tool (Yale University). Patient-specific data points captured from the RDPH electronic medical record included admission, demographics, and comorbidities, as well as microbiological, biochemical, clinical, and outcome data. Clinical episode details included the presenting clinical syndrome and several preexisting comorbidities (see supplementary methods for specific definitions).

Some isolates were deemed to represent colonization by the attending clinician and therefore not treated. These patients were included to assess for differences in epidemiology or outcomes as compared with isolates judged pathogenic and requiring antimicrobial therapy; for clarity, they are grouped as an additional “infection” syndrome. We were specifically interested in a history of water exposure and type. Patients were grouped by water exposure and salinity (defined as electronic medical record documentation of water exposure, including salinity if specified), no water exposure (documented lack of water exposure), and unknown water exposure (where no documentation of exposure was made). Information on associated injuries or trauma was also collected.

Laboratory markers captured were the initial white cell count (cells × 10^9^/L) and the highest C-reactive protein (milligrams per liter) ≤72 hours from the index microbiology. Organism-specific details recorded include sites of isolation and the isolation of other (non-VACS) organisms. Treatment data were recorded, including initial and subsequent antibiotic therapy and the nature and extent of surgical management.

Clinical outcomes collected were the presence of sepsis or shock (as judged by the admitting clinician), intensive care unit (ICU) admission and length of stay (LoS), infection-specific death, ≤ 90-day all-cause mortality, hospital LoS, and infection-related readmission. Infection-related readmission was defined as representation due to either nonresolving local symptoms or new systemic features of infection (ie, fever) within 90 days of the index microbiology.

Given that there are no published Clinical & Laboratory Standards Institute break points for these organisms, we considered *Vibrio* spp isolates susceptible to ceftazidime, ceftriaxone, ciprofloxacin, and doxycycline; *Aeromonas* spp to ciprofloxacin, doxycycline, meropenem, piperacillin-tazobactam, and trimethoprim-sulfamethoxazole; *C violaceum* to ciprofloxacin, doxycycline, meropenem, and trimethoprim-sulfamethoxazole; and *Shewanella* spp to ceftazidime, ciprofloxacin, gentamicin, meropenem, and trimethoprim-sulfamethoxazole [13]. Receipt of an antibiotic effective against the isolated VACS species was considered “VACS-effective therapy.” Individual isolate resistance profiles were not matched to patient-specific antimicrobial prescription. We assessed and categorized time frames for prescription of VACS-effective therapy into ≤72 hours, ≤7 days, or any exposure from the date of the index microbiology. We did not stratify by dosing regimen or therapeutic drug levels, only whether the antimicrobials were prescribed or not.

Copathogens were recorded if they were isolated from the same site as the VACS organism. These were grouped into *S aureus*, *Streptococcus* spp, enteric flora, pseudomonads, other, or none (supplementary methods).

Statistical analyses were performed with Stata version 17 (StataCorp). Binary and categorical variables were compared via Pearson χ^2^ test and continuous variables by the Kruskal-Wallis test. Post hoc analysis of categorical variables with a Fisher exact approach was performed if initial testing met significance criteria (*P* ≤ .05) as described [15]. Tables were created by the Stata module “table1_mc” [16].

### Patient Consent Statement

This study was registered with the Human Research Ethics Committee of the Northern Territory Department of Health and Menzies School of Health Research (reference 2023-45), and the requirement for informed consent was waived due to the retrospective and observational study design.

## RESULTS

**Figure 1.**
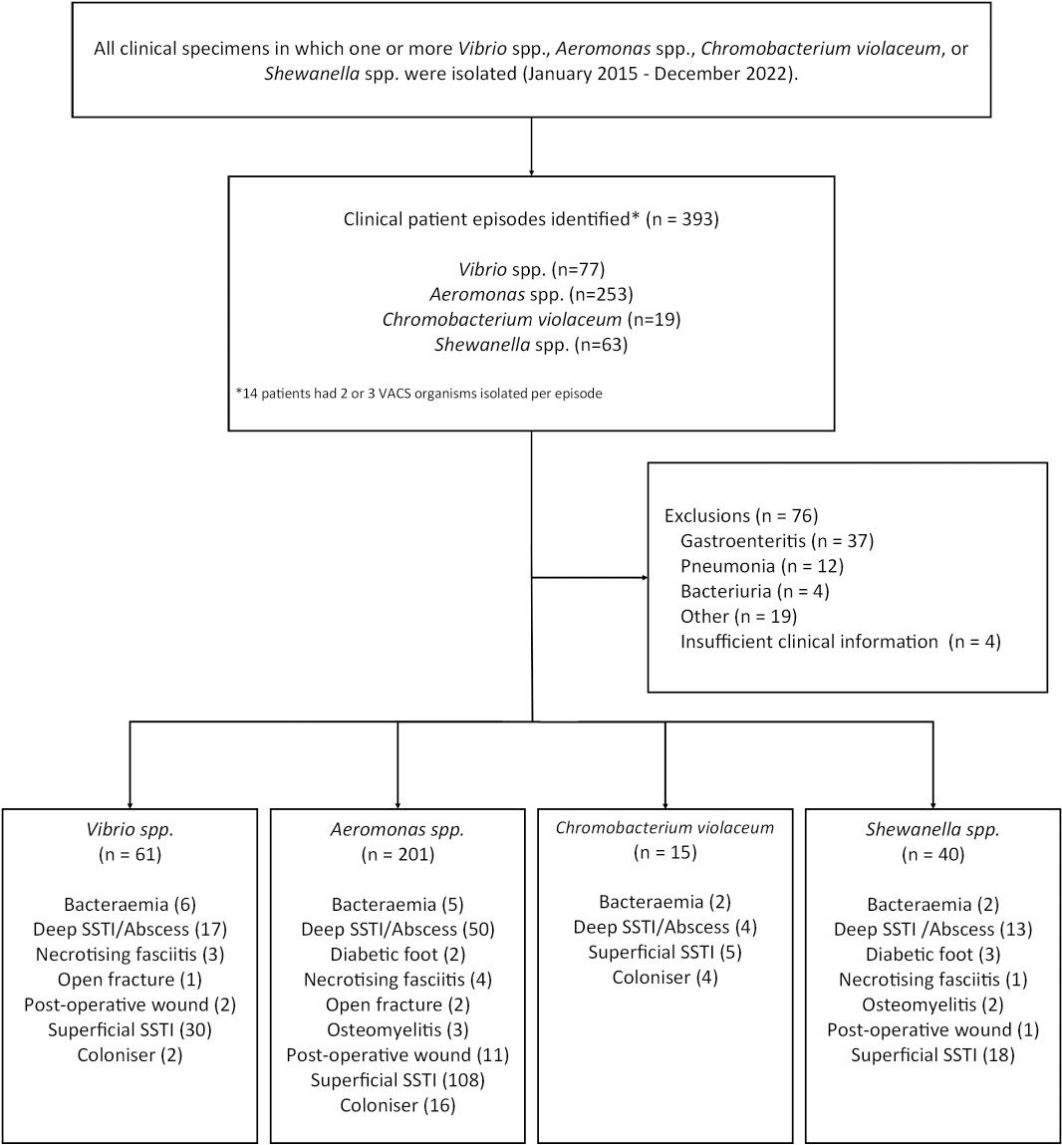
Flowchart describing the number of patients included and excluded from analysis, grouped by species. SSTI, skin and soft tissue infection; VACS, *Vibrio* spp, *Aeromonas* spp, *Chromobacterium violaceum*, and *Shewanella* spp.

We identified 393 patient episodes where VACS organisms were isolated, 76 of which were excluded for reasons as described in Figure 1, resulting in 317 episodes included in the study, which made up ∼0.7% of all gram-negative organism isolations over the study period. Patient demographic and comorbidity data are shown in Table 1. The most commonly isolated organism was *Aeromonas* spp (201/317, 63.4%), followed by *Vibrio* spp (61/317, 19.2%), *Shewanella* spp (40/317, 12.6%), and *C violaceum* (15/317, 4.7%). There was a marked male preponderance (236/317, 74.4%), which was similar among VACS groups (*P* = .12). Indigenous (First Nations) Australians accounted for 119 (38.0%) patients. The majority of infections (243/317, 76.7%) were managed at RDPH, followed by GDH (41/317, 12.9%) and KDH (29/317, 9.1%). Assessment of the interaction between organism and site of isolation was investigated with an assumed null hypothesis of there being no difference in the relative distribution of infections. This was not the case, with post hoc testing finding the rates of *Aeromonas* spp isolation lower than expected at GDH (*P* = .003) but higher in KDH (*P* = .002). Conversely, *Vibrio* spp were isolated at lower rates from KDH (*P* = .004) and higher at GDH (*P* = .02).

**Table 1.** Demographic Data of Patients With VACS Bacteria Isolated in the Top End From Deep and Superficial Infections: 2015–2022

	*Vibrio* spp	*Aeromonas* spp	*C violaceum*	*Shewanella* spp	Total	*P* Value
Total	61 (19)	201 (63)	15 (5)	40 (13)	317	
Sex						.12
Male	49 (80.3)	144 (71.6)	9 (60.0)	34 (85.0)	236 (74.4)	
Female	12 (19.7)	57 (28.4)	6 (40.0)	6 (15.0)	81 (25.6)	
Age, y, median (IQR)	37.0 (26.1–52.8)	41.5 (25.1–56.3)	46.4 (32.0–54.6)	45.8 (37.7–54.7)	42.2 (26.1–55.3)	.22
<18	5 (8.2)	29 (14.4)	2 (13.3)	1 (2.5)	37 (11.7)	
18–55	43 (70.5)	117 (58.2)	10 (66.7)	29 (72.5)	206 (65.0)	
>55	13 (21.3)	55 (27.4)	3 (20.0)	10 (25.0)	81 (25.6)	
Ethnicity^a^						.069
Indigenous Australians	21 (34.4)	81 (40.3)	1 (6.7)	16 (40.0)	119 (37.5)	
Non-Indigenous Australians	38 (65.6)	119 (59.7)	14 (92.3)	23 (60)	194 (62.5)	
Location						<.001
RDPH	42 (68.9)	158 (78.6)	13 (86.7)	30 (75.0)	243 (76.7)	
GDH	16 (26.2)^b^	17 (8.5)^b^	0 (0.0)	8 (20.0)	41 (12.9)	
KDH	0 (0.0)^b^	26 (12.9)^b^	2 (13.3)	1 (2.5)	29 (9.1)	
Other	3 (4.9)^b^	0 (0.0)	0 (0.0)	1 (2.5)	4 (1.3)	
Comorbidities						
Cigarette use	9 (14.8)	55 (27.4)	2 (13.3)	5 (12.5)	71 (22.4)	.049
Chronic kidney disease	11 (18.0)	42 (20.9)	0 (0.0)	10 (25.0)	63 (19.9)	.2
Diabetes	5 (8.2)	30 (14.9)	4 (26.7)	6 (15.0)	45 (14.2)	.28
Alcohol excess	3 (4.9)	21 (10.4)	0 (0.0)	3 (7.5)	27 (8.5)	.33
Chronic liver disease	3 (4.9)	17 (8.5)	2 (13.3)	3 (7.5)	25 (7.9)	.69
Obesity	4 (6.6)	9 (4.5)	1 (6.7)	2 (5.0)	16 (5.0)	.92
Immunocompromise	3 (4.9)	6 (3.0)	3 (20.0)	1 (2.5)	13 (4.1)	.014
Chronic lung disease	4 (6.6)	5 (2.5)	0 (0.0)	4 (10.0)	13 (4.1)	.092
Heart failure	1 (1.6)	8 (4.0)	0 (0.0)	4 (10.0)	13 (4.1)	.16
Previous intravenous drug use	0 (0.0)	7 (3.5)	0 (0.0)	2 (5.0)	9 (2.8)	.36
Hemodialysis	1 (1.6)	5 (2.5)	0 (0.0)	0 (0.0)	6 (1.9)	.69
Water exposure						<.001
None	11 (18.0)^b^	144 (71.6)^b^	11 (73.3)	22 (55.0)	188 (59.3)	
Salt water	30 (49.2)^b^	3 (1.5)^b^	0 (0.0)	14 (35.0)^b^	47 (14.8)	
Freshwater	4 (6.6)	26 (12.9)	3 (20.0)	0 (0.0)^b^	33 (10.4)	
Unknown exposure	14 (23.0)^b^	18 (9.0)	0 (0.0)	0 (0.0)	32 (10.1)	
Unknown salinity	2 (3.3)	6 (3.0)	0 (0.0)	4 (10.0)	12 (3.8)	
Brackish water	0 (0.0)	4 (2.0)	1 (6.7)	0 (0.0)	5 (1.6)	

Data are presented as No. (%) unless noted otherwise.

Abbreviation: *C violaceum*, *Chromobacterium violaceum*; GDH, Gove District Hospital; KDH, Katherine District Hospital; RDPH, Royal Darwin and Palmerston hospitals; VACS, *Vibrio* spp, *Aeromonas* spp, *Chromobacterium* violaceum, and *Shewanella* spp.

^a^Ethnicity data missing for 4 records.

^b^ *P* < .05 in Fisher exact post hoc analysis.

The most common comorbidities were cigarette use (71/317, 22.4%), chronic kidney disease (63/317, 19.9%), diabetes mellitus (45/317, 14.2%), and alcohol excess (27/317, 8.5%). Cigarette use was associated with *Aeromonas* spp isolation (*P* = .049), while immunocompromise was associated with *C violaceum* (*P* = .014). Most infections were not associated with documented water exposure (188/317, 59.3%). In those with documented water exposure, saltwater exposure was most common (47/96, 50.0%). Saltwater exposure was proportionally most associated with either *Vibrio* spp (30/61, 49.2%) or *Shewanella* spp (14/40, 35.0%) infections, though neither was significant in post hoc testing. *Shewanella* spp isolation was less frequent in freshwater exposures (*P* = .02). The suspected etiology of non–water-associated SSTIs included trauma (88/188, 46.8%), bite injuries (14/188, 7.4%), and postoperative wound infections (14/188, 7.4%). Post hoc analysis identified trauma most commonly associated with *Aeromonas* spp (*P* < .001) and least with *Shewanella* spp (*P* < .001). Bites were predominately due to dogs (5/14, 35.7%) or insects (4/14, 28.6%) but also included snake, horse, human, and cat (each associated with a single episode; another was unspecified).

The clinical syndromes identified, site of infection, and laboratory data are summarized in Table 2. Superficial SSTI was the most common clinical syndrome, with 161 of 317 episodes (50.8%), followed by deep SSTI/abscess (84/317, 26.5%). Eight (2.5%) NF syndromes were recorded, 50% of which occurred in the *Aeromonas* spp cohort (4/8, 50%). NF, as a proportion of total infections, was greatest in the *Vibrio* spp group (3/61, 4.9%). Diabetic foot infections were uncommon, identified in only 5 of 317 (1.6%) episodes. Fifteen (4.7%) patients had bacteremia documented, the highest proportions seen with *C violaceum* (2/15, 13.3%) and *Vibrio* spp (6/62, 9.8%) infections. The relative lack of bacteremia in the *Aeromonas* spp cohort was statistically significant (*P* = .016). *C violaceum* was most associated with colonization (4/15, 26.7%; *P* = .014).

**Table 2.** Clinical Data of Patients With VACS Organisms Isolated From Deep and Superficial Infections in the Top End: 2015–2022

	*Vibrio* spp	*Aeromonas* spp	*C violaceum*	*Shewanella* spp	Total	*P* Value
Total	61 (19)	201 (63)	15 (5)	40 (13)	317 (100)	
Clinical syndrome						.013
Superficial SSTI	30 (49.2)	108 (53.7)	5 (33.3)	18 (45.0)	161 (50.8)	
Deep SSTI or abscess	17 (27.9)	50 (24.9)	4 (26.7)	13 (32.5)	84 (26.5)	
VACS organism colonizer	2 (3.3)	16 (8.0)	4 (26.7)^a^	0 (0.0)	22 (6.9)	
Bacteremia	6 (9.8)	5 (2.5)^a^	2 (13.3)	2 (5.0)	15 (4.7)	
Postoperative wound	2 (3.3)	11 (5.5)	0 (0.0)	1 (2.5)	14 (4.4)	
Necrotising fasciitis	3 (4.9)	4 (2.0)	0 (0.0)	1 (2.5)	8 (2.5)	
Osteomyelitis	0 (0.0)	3 (1.5)	0 (0.0)	2 (5.0)	5 (1.6)	
Diabetic foot infection	0 (0.0)	2 (1.0)	0 (0.0)	3 (7.5)^a^	5 (1.6)	
Open fracture	1 (1.6)	2 (1.0)	0 (0.0)	0 (0.0)	3 (0.9)	
Site of infection^b^						<.001
Foot	23 (37.7)	71 (35.3)	1 (6.7)^a^	15 (37.5)	110 (34.7)	
Leg	18 (29.5)	65 (32.3)	5 (33.3)	16 (40.0)	104 (32.8)	
Hand	12 (19.7)	21 (10.4)	1 (6.7)	5 (12.5)	39 (12.3)	
Torso	0 (0.0)^a^	14 (7.0)	2 (13.3)	0 (0.0)	16 (5.0)	
Arm	2 (3.3)	12 (6.0)	1 (6.7)	0 (0.0)	15 (4.7)	
Groin	1 (1.6)	6 (3.0)	0 (0.0)	1 (2.5)	8 (2.5)	
Head	0 (0.0)	6 (3.0)	1 (6.7)	1 (2.5)	8 (2.5)	
Nonwound	3 (4.9)	2 (1.0)	1 (6.7)	1 (2.5)	7 (2.2)	
Unknown	1 (1.6)	2 (1.0)	3 (20.0)^a^	0 (0.0)	6 (1.9)	
Multiple	1 (1.6)	2 (1.0)	0 (0.0)	1 (2.5)	4 (1.3)	
Coinfections						
Enteric bacteria	21 (34.4)	134 (66.7)	2 (13.3)	23 (57.5)	180 (56.8)	<.001
*Staphylococcus aureus*	29 (47.5)	94 (46.8)	4 (26.7)	25 (62.5)	152 (47.9)	.1
Other	27 (44.3)	103 (51.2)	2 (13.3)	14 (35.0)	146 (46.1)	.014
*Streptococcus* spp	14 (23.0)	41 (20.4)	3 (20.0)	20 (50.0)	78 (24.6)	.001
None	13 (21.3)	21 (10.4)	6 (40.0)	2 (5.0)	42 (13.2)	<.001
Pseudomonads	1 (1.6)	18 (9.0)	2 (13.3)	4 (10.0)	25 (7.9)	.22
Laboratory,^c^ median (IQR)						
WCC, cells × 10^9^/L	9.9 (8.4–13.5)	10.9 (8.8–14.7)	12.8 (11.1–14.4)	10.9 (7.7–15.3)	10.9 (8.6–14.7)	.2
CRP, mg/L	25.3 (5.3–62.0)	31.2 (12.2–114.8)	174.0 (40.0–294.7)	68.0 (17.0–178.0)	35.1 (12.8–132.0)	.056

Data are presented as No. (%) unless noted otherwise.

Abbreviations: *C violaceum*, *Chromobacterium violaceum*; CRP, C-reactive protein; SSTI, skin and soft tissue infection; VACS, *Vibrio* spp, *Aeromonas* spp, *Chromobacterium violaceum*, and *Shewanella* spp; WCC, white cell count.

^a^ *P* < .05 in Fisher exact post hoc analysis.

^b^Four patients (2 *Aeromonas*, 1 *Shewanella*, and 1 *Vibrio*) had multiple sites of infection; all sites have been included.

^c^Where recorded.

Coinfections with other recognized pathogenic bacteria were common. Coisolation of *S aureus* and enteric bacteria was seen in 47.9% (152/317) and 56.8% (180/317), respectively. Of 317 episodes, 42 (13.2%) were monomicrobial infections, most commonly in *C violaceum* (6/15, 40%; *P* < .001).

Infections typically involved the lower limb, with foot and leg infections accounting for approximately a third of isolations each (110/317 [34.7%] and 104/317 [32.8%], respectively). The proportion of infections of the hand was highest in the *Vibrio* spp cohort (12/61, 19.7%), whereas infection of the lower limb (foot and leg) was highest in the *Shewanella* spp group (31/40, 77.5%), though neither reached statistical significance individually (specific body part) or when grouped as upper limb (hand and arm) or lower limb (foot and leg).

Table 3 identifies the VACS bacterial species isolated in each group. Nonspeciated *Aeromonas* species was the most common organism group identified (183/333, 55%). *S algae* was the predominant *Shewanella* isolate (46/52, 88.5%). *V alginolyticus* (26/61, 42.6%), *V parahaemolyticus* (20/61, 32.8%), and *V vulnificus* (6/61, 9.8%) accounted for the majority of *Vibrio* spp speciation.

**Table 3.** Speciation of VACS Bacteria Isolated in the Top End From Deep and Superficial Infections: 2015–2022

Species	No. (%)^a^
*Vibrio*	
*V alginolyticus*	26 (7.8)
*V cholerae*	1 (0.3)
*V fluvialis*	2 (0.6)
*V mimicus*	1 (0.3)
*V parahaemolyticus*	20 (6.0)
*V vulnificus*	6 (1.8)
Multiple species	5 (1.5)
*Aeromonas*	
Species	183 (55.0)
*A hydrophilia*	9 (2.7)
*A sobrica*	12 (3.6)
Multiple species	1 (0.3)
*Chromobacterium violaceum*	15 (4.5)
*Shewanella*	
*S algae*	46 (13.8)
*S putrefaciens*	5 (1.5)
Species	1 (0.3)
Total	333

Abbreviation: VACS, *Vibrio* spp, *Aeromonas* spp, *Chromobacterium violaceum*, and *Shewanella* spp.

^a^14 patients had ≥2 species detected from the wound—predominantly *Vibrio* spp/*Shewanella* spp and *Vibrio* spp/*Aeromonas* spp.

Table 4 details the antimicrobial therapy and outcomes of the cohort. Just under half of patients (149/317, 47.0%) received VACS-effective empiric antimicrobial therapy within 72 hours of the index culture. Therapy between 72 hours and 7 days postisolation was VACS effective in just over half of patients (163/317, 51.4%), and 47.0% (149/317) received no VACS-effective therapy at any point during the index admission. The receipt of VACS-effective empirical antibiotics was associated with ICU admission (13/15 admissions, 86.7%; *P* = .002). In total, 41 (13%) of 317 patients were readmitted with a complication of the index infection. A lack of VACS-effective antimicrobial therapy was not associated with readmission, as 23 (56.1%) of 41 patients readmitted had VACS-effective therapy over any time frame (*P* = .67).

**Table 4.** Outcome Data of Patients With VACS Organisms Isolated From Deep and Superficial Infections in the Top End: 2015–2022

	*Vibrio* spp	*Aeromonas* spp	*C violaceum*	*Shewanella* spp	Total	*P* Value
Total	61 (19)	201 (63)	15 (5)	40 (13)	317 (100)	
Medical treatment ^a^						
Antibiotics ≤72 h	36 (59.0)	93 (46.3)	9 (60.0)	12 (30.0)	150 (47.3)	.03
Antibiotics ≤7 d	37 (60.7)	101 (50.2)	9 (60.0)	17 (42.5)	164 (51.7)	.27
Any effective antibiotics	37 (60.7)	105 (52.2)	9 (60.0)	18 (45.0)	169 (53.3)	.42
Surgical data						
No. of operations						.33
0	43 (70.5)	109 (54.2)	10 (66.7)	27 (67.5)	189 (59.6)	
1	15 (24.6)	55 (27.4)	4 (26.7)	11 (27.5)	85 (26.8)	
2	0 (0.0)	16 (8.0)	1 (6.7)	1 (2.5)	18 (5.7)	
3	0 (0.0)	4 (2.0)	0 (0.0)	0 (0.0)	4 (1.3)	
4	1 (1.6)	5 (2.5)	0 (0.0)	0 (0.0)	6 (1.9)	
5	1 (1.6)	0 (0.0)	0 (0.0)	0 (0.0)	1 (0.3)	
>5	1 (1.6)	12 (6.0)	0 (0.0)	1 (2.5)	14 (4.4)	
Required skin graft	3 (4.9)	20 (10.0)	0 (0.0)	1 (2.5)	24 (7.6)	.18
Required amputation	0 (0.0)	8 (4.0)	0 (0.0)	1 (2.5)	9 (2.8)	.36
Severity						
Shock	4 (6.6)	5 (2.5)	2 (13.3)	1 (2.5)	12 (3.8)	.11
ICU admission	4 (6.6)	9 (4.5)	2 (13.3)	0 (0.0)	15 (4.7)	.18
Length of stay, d, median (IQR)	2.0 (0.0–5.0)	3.0 (0.0–9.0)	2.0 (0.0–14.0)	3.0 (0.0–6.0)	3.0 (0.0–7.0)	.90
Readmitted	5 (8.2)	27 (13.4)	2 (13.3)	7 (17.5)	41 (12.9)	.57
Infection-related ≤90-d mortality	1 (1.6)	4 (2.0)	1 (6.7)	2 (5.0)	8 (2.5)	.48
Discharge type						<.001
Planned	37 (60.7)	166 (82.6)	13 (86.7)	31 (77.5)	247 (77.9)	
Not specified	15 (24.6)	18 (9.0)	1 (6.7)	0 (0.0)	34 (10.7)	
Self-directed	5 (8.2)	5 (2.5)	1 (6.7)	9 (22.5)	20 (6.3)	
Transferred	3 (4.9)	10 (5.0)	0 (0.0)	0 (0.0)	13 (4.1)	
Died in hospital	1 (1.6)	2 (1.0)	0 (0.0)	0 (0.0)	3 (0.9)	

Receipt of effective antibiotics within 72 hours or 7 days of the index microbiology. Data are presented as No. (%) unless noted otherwise.

Abbreviations: *C violaceum*, *Chromobacterium violaceum*; ICU, intensive care unit; VACS, *Vibrio* spp, *Aeromonas* spp, *Chromobacterium violaceum*, and *Shewanella* spp.

^a^Effective antimicrobial therapy was defined as follows: *Vibrio* spp isolates were considered susceptible to ceftazidime, ceftriaxone, ciprofloxacin, and doxycycline; *Aeromonas* spp to ciprofloxacin, doxycycline, meropenem, piperacillin-tazobactam, and trimethoprim-sulfamethoxazole; *C violaceum* to ciprofloxacin, doxycycline, meropenem, and trimethoprim-sulfamethoxazole; and *Shewanella* spp to ceftazidime, ciprofloxacin, gentamicin, meropenem, and trimethoprim-sulfamethoxazole.

There was a statistically significant association between exposure type and the receipt of VACS-effective empiric antibiotics (Supplementary Table 1; *P* < .001). VACS-effective empirical antibiotics were prescribed in 68.1% (32/47, *P* = .002) of saltwater exposures and 69.7% (24/33, *P* = .003) of freshwater exposures but in only 37.8% (71/188, *P* < .001) of non–water-exposed isolates.

Surgical procedures were performed in 128 (40.4%) of 317 patients, with the highest proportion (92/201, 45.8%) in patients with *Aeromonas* spp infections, though there were no significant differences in group proportions for surgical procedures. Of 317 patients, 24 (7.6%) required skin grafting, of which 20 (83.3%) were associated with *Aeromonas* spp isolation. Of 128 patients, 9 (7.0%) required amputations: 8 (88.9%) in the *Aeromonas* spp group and 1 (11.1%) in the *Shewanella* spp group, though this association was nonsignificant (*P* = .364).

In total, 15 (4.7%) of 317 patients were admitted to the ICU, and 3 (0.9%) died in the hospital due to their infection. Few patients admitted to the ICU (4/15, 26.7%) had documented water exposure. Most ICU admissions were from the *Aeromonas* spp group (9/15, 60.0%). The majority of clinical syndromes of those admitted to the ICU were superficial SSTI, NF, and bacteremia, all in 4 of 15 patients (26.7%), though only bacteremia and NF were statistically overrepresented (*P* = .003 and *P* < .001, respectively). Of those admitted to the ICU, 7 (46.7%) presented with shock. A lack of any copathogen was associated with ICU admission (*P* = .019). For those admitted to the ICU, the median LoS was 4 days (IQR, 2–11) with an overall hospital LoS of 28 days (IQR, 12–55). The median LoS for the overall cohort was 3 days (IQR, 0–7).

In addition to the 3 infection-related deaths in the hospital, there were 5 deaths ≤90 days from the index microbiology, making overall mortality 2.5% (8/317). Half of the fatalities (4/8, 50.0%) were in the *Aeromonas* spp group, and only 1 (12.0%) of 8 fatalities had documented water exposure. The primary clinical syndrome associated with the deaths was bacteremia (3/8, 37.5%), NF (2/8, 25.0%), colonization (2/8, 25.0%), and deep SSTI/abscess (1/8, 12.5%).

The incidence and outcomes of colonizer isolates were compared with the pathogenic group (Supplementary Table 2). Colonizers were associated with higher rates of readmission (*P* = .038) and 90-day mortality (*P* = .042) but not with any change in copathogen rates. For colonizers, there were differences in site of isolation (groin overrepresented, *P* = .013), reduced VACS-effective antibiotic exposure (*P* = .003), and overrepresentation of some comorbidities (chronic lung disease, *P* = .001; heart failure, *P* = .019; hemodialysis, *P* = .010; diabetes, *P* = .014).

## DISCUSSION

This study reviews the spectrum of VACS organisms associated with deep and superficial infections from a single tropical health service over an 8-year span. The broadly similar patient demographics, comorbidities, and pattern of clinical syndromes suggest that the previously ascribed acronym “VACS” has merit in terms of VACS-associated infection [13]. Although other marine-associated infections are well described (eg, *Edwardsiella tarda*, *Erysipelothrix rhusiopathiae*), the VACS group shares several clinical features [2]. Other studies have compared some but not all the bacteria, and previous reviews often examined individual species or case reports [9, 12, 17, 18].

The marked adult male predominance and few pediatric cases seen in this study were significant and are consistent with findings of previous studies of VACS SSTI [13, 18]. This male predominance, however, is not a feature of other skin infections, such as community-acquired methicillin-resistant *S aureus* [18]. This may reflect a combination of factors, including exposure-prone occupational and recreational activities and comorbidities such as alcohol use disorder [19]. Estrogen is postulated to play a protective role against the effects of *V vulnificus* endotoxin [20].

Indigenous (First Nations) Australians, especially in central and northern Australia, are often disproportionally affected by infectious diseases. Although there were no species-specific associations with ethnicity, 38% of the cohort identified as Indigenous, which is higher than the Indigenous population rate in our region of 30% [21]. This may reflect in part the rural and remote residences of many Indigenous people of the Northern Territory, with close links to the environment in daily activities. The higher proportion of *Aeromonas* spp from KDH likely reflects its inland geography, while the higher proportion of *Vibrio* spp at GDH is consistent with the coastal locale, where Indigenous Australians identify as “saltwater people.”

The levels of chronic diseases seen in this cohort are moderately higher than those of the Northern Territory adult population overall: we found increased rates of chronic kidney disease and hemodialysis (21.8% vs 9.4%), diabetes (14.2% vs 9.1%), and chronic lung disease (4.1% vs 2.9%) in our study vs baseline rates [22]. Given the known association between *Vibrio* spp and *Shewanella* spp with diabetes and chronic kidney disease, this could have led to an overrepresentation of these organisms; however, *Aeromonas* spp were still by far the predominant organism in our cohort.

Although all VACS organisms are known to be water associated, <40% in this cohort had documented water exposure (10.1% had unknown exposure). *Aeromonas* spp infections are associated with the wet season and can be more ubiquitous in the environment [13, 18]. However, a considerable number of *Vibrio* spp and *Shewanella* spp isolations did not have documented water exposure (18% and 55%, respectively) despite these organisms being strongly associated with salt water [2]. This could be a result of the lack of inquiry or documentation regarding water exposure but could also represent an underrecognized presence of these organisms in nonaquatic environments. Climate change and associated greater water temperatures are projected to increase the distribution of *Vibrio* spp, markedly raising the populations exposed to this pathogen [23]. This highlights the need for clinical consideration of these organisms in not only our tropical environment but other novel settings, particularly with trauma in patients with comorbidities. This study contributes contemporary information about *Vibrio* spp infections to a wider audience who may be increasingly confronted with such presentations as climate change impacts on microbial ecology.

Due to the previously described associations between VACS organisms and poor outcomes, our institution has produced specific guidelines for empiric treatment of wounds associated with water exposure. Therefore, these guidelines are likely responsible for some of the association between documented water exposure and VACS-effective empirical treatment seen in our study.

For this study, our definition of colonizers was simply those VACS organisms managed as such in the clinical records by the attending clinician. Our data suggest that a potentially even larger number of VACS organisms may have been “colonizers,” though not explicitly documented as such. This particularly applies to scenarios where there was coisolation of *S aureus* and/or *Streptococcus* spp and where clinical improvement occurred despite empirical therapy without a VACS-effective antimicrobial. Given that culture results may not be available within the first 48 hours, the decision to switch to VACS-effective therapy on isolation of a VACS organism requires clinical judgment. These high rates of copathogen isolation make it difficult to ascribe the specific virulence or pathogenicity of each isolate. While the isolated VACS may be a colonizer in those already clinically improving, we now recommend consideration of VACS-effective therapy for those with more severe infections or significant comorbidities, who are at higher risk of subsequent readmission and mortality, irrespective of water exposure history.

One group of patients who had adequate levels of VACS-effective empirical therapy, despite little documented water exposure, comprised those admitted to the ICU where increased severity of illness prompts expansion of antimicrobial coverage. This is typically meropenem in our ICU due to the high rates of melioidosis in our region (due to *Burkholderia pseudomallei*) [24]. Of note, *B pseudomallei* can itself result in water-associated infections [25]. Following initial intravenous therapy in the ICU, rationalization of subsequent antimicrobial therapy can be guided by whether the organisms isolated are VACS, *B pseudomallei*, or others, including community-acquired methicillin-resistant *S aureus*, which is common in our setting [26].

Postoperative wound infections with *Aeromonas* spp are rare, with only 21 reported cases worldwide until 2009 [27]. However, we identified 11 patients with *Aeromonas* spp from postoperative wound specimens. This is unlikely to reflect exposure to and infection with *Aeromonas* spp postoperatively but rather prior environmental exposure of a partially healed wound with culture of *Aeromonas* spp after surgery, usually in an individual with multiple comorbidities.

There are several limitations to this study. As a retrospective study, it relies on the complete and accurate clinical documentation of the treating teams at the time of admission. Considering our results, this limitation is especially relevant to the documentation or not of water exposure. Occult water exposure that is unrecognized by the patient or deemed insufficient to meet an “exposure threshold” by the treating clinician is possible. To better analyze this, prospective studies should ensure adequate recording of water type and specific exposure to allow better assessments of VACS infection risk. Similar issues surround the classification of colonizer VACS isolates, which were documented in only a small proportion of patients. These and other data points relied on the assessment of the treating team, which is made on a case-by-case basis with clinicians possibly applying varied definitions of syndrome, exposure type, or mechanisms of injury. In areas where these infections are more commonly seen, we recommend specific documentation of the presence of water exposure or lack thereof.

Furthermore, due to the lack of published break points and large number of species with each group, we assumed sensitivity of broad classes of antimicrobials to each category of organisms. The large number of copathogen isolations also makes it difficult to precisely determine the specific pathology related to the isolated VACS organism and hence challenging to comment on specific causality.

In summary, VACS group infections have similar within-group risk factors, clinical manifestations, and outcomes. The isolation of a VACS organism in our setting was often not associated with documented water exposure, and severe disease and death were uncommon. Despite this, we recommend treatment with appropriate antimicrobials should these organisms be isolated.

## Supplementary Material

ofae319_Supplementary_Data

## Supplementary Data

Supplementary materials are available at *Open Forum Infectious Diseases* online. Consisting of data provided by the authors to benefit the reader, the posted materials are not copyedited and are the sole responsibility of the authors, so questions or comments should be addressed to the corresponding author.

## Notes

***Author contributions*.** Study conceptualization and design: K. M., R. W. B., B. J. C., S. V., S. J. Data acquisition: K. M., S. C., E. L. S., T. K. Data analysis: S. C., R. W. B., S. V., B. J. C., E. L. S. Initial drafting: S. C., R. W. B., S. V., K. M., T. K. Final drafting: S. C., K. M., T. K., E. L. S., S. J., B. J. C., S. V.

***Financial support***. This work was not specifically funded as it was undertaken as part of routine clinical audit.
