# Supplementary material for: Clinical Presentation and Outcomes Following Infection With Vibrio spp, Aeromonas spp, Chromobacterium violaceum, and Shewanella spp Water-Associated Organisms in Tropical Australia, 2015–2022
Source: Open Forum Infect Dis. 2024 Jun 12;11(7):ofae319. doi: 10.1093/ofid/ofae319 (PMC11227229; doi:10.1093/ofid/ofae319)
Supplement: ofae319_Supplementary_Data [file ofae319_supplementary_data.docx]

Supplementary Material to the manuscript titled:

**“Clinical presentation and outcomes following infection with Vibrio spp., Aeromonas spp., Chromobacterium violaceum and Shewanella spp. (VACS) water-associated organisms in tropical Australia, 2015-2022”**

**Supplementary Methods**

Comorbidities were defined as follows:

1. Alcohol excess
   1. As per the Australian clinical guidelines (NHMRC) or if alcohol excess or similar was documented by the attending clinician.
2. Cigarette use
   1. Any cigarette use in the preceding 7 days of admission or if smoking/smoker was listed in admission documentation.
3. Chronic lung disease
   1. The presence of previously diagnosed asthma, bronchiectasis, chronic obstructive pulmonary disease, or interstitial lung disease documented in the EMR.
4. Chronic Liver Disease
   1. Defined as a previous diagnosis of cirrhosis (any grade) or hemochromatosis.
5. Chronic Kidney Disease
   1. Defined as an estimated glomerular filtration rate of <60mL/min but not receiving any form of renal replacement therapy.
6. Heart failure
   1. Any documented history of heart failure, either with preserved or reduced ejection fraction documented in admission notes.
7. Haemodialysis
   1. The receipt of any haemodialysis for any indication in the preceding 4 weeks.
8. Immunocompromise
   1. As per the Australian Technical Advisory Group on Immunisation (ATAGI) guidelines – Box one available at ([www.health.gov.au/sites/default/files/2024-01/atagi-recommendations-on-the-use-of-a-third-primary-dose-of-covid-19-vaccine-in-individuals-who-are-severely-immunocompromised.pdf](http://www.health.gov.au/sites/default/files/2024-01/atagi-recommendations-on-the-use-of-a-third-primary-dose-of-covid-19-vaccine-in-individuals-who-are-severely-immunocompromised.pdf)).
9. Diabetes mellitus
   1. Defined as either the receipt of either oral or injectable anti-hyperglycaemic agent or a HbA1c of >7%.
10. Obesity
    1. As per the documentation by the attending clinician.
11. Injecting drug use
    1. The use of any non-prescribed substance used intravenously in the preceding 12 months.

Clinical syndromes were listed as per the admitting or discharge diagnosis as documented by the attending clinician, and then aggregated as follows. Clinical syndromes included in analysis were:

1. Superficial SSTI
   1. Cellulitis, erysipelas, traumatic abrasions, or cuts not extending deeper then subcutaneous tissue and not requiring any stitching for closure.
2. Deep SSTI/abscess
   1. Infection or wound extends deeper than subcutaneous tissue (e.g. muscle, ligament/tendons), or if there exists an abscess that required drainage (either bedside or in the operating theatre). Typically, this syndrome required some form of mechanical closure (predominately stitches).
3. Necrotising skin and soft tissue infection
   1. As adjudged by the attending clinician.
   2. Our institution typically assesses for the clinical features of Necrotising fasciitis as documented by the eTG “Diagnosing necrotising skin and soft tissue infections” guideline.
4. Diabetic Foot Infection
   1. Defined as per the “IWGDF/IDSA Guidelines on the Diagnosis and Treatment of Diabetes-related Foot Infections”.
   2. IE: The presence of an ulcer associated with two or more of local swelling or induration, erythema extending more than 0.5 cm in any direction from the wound, local tenderness or pain, local warmth, or purulent discharge.
5. Bacteraemia of uncertain source
   1. Isolation of a VACS organisms from one or more blood cultures.
   2. This superseded previous categories such that if an organism was isolated from both an abscess and blood culture, this would count towards Bacteraemia but not deep SSTI/abscess.
6. Coloniser
   1. Isolation of the organism in a clinical specimen with documented clinical opinion that this was not contributing to the pathogenesis of the infection.

Clinical syndromes excluded were:

1. Pneumonia
   1. Isolation of an organism from sputum or another site with radiological and clinical evidence of pneumonia.
2. Gastroenteritis
   1. Isolation of an organism from faecal samples without a concomitant syndrome meeting an inclusion criterion.
3. Urinary tract infection
   1. Defined as isolation of an organism from urine without a concomitant syndrome meeting an inclusion criterion.
4. Primary intra-abdominal infection
   1. Defined as cholangitis, cholecystitis, peritonitis, pyelonephritis.
5. All other isolates not obtained from a site of primary trauma.

Co-pathogen definitions

1. Staphylococcus aureus
   1. Both methicillin sensitive and resistant isolates were grouped.
2. Streptococcal species
   1. This includes the aerobic and anaerobic species, and all Lancefield grouping types.
3. Enteric flora
   1. This included also Enterobacteriaceae, coliforms, bowel flora such as Enterococcal species.
4. Pseudomonads
   1. This encompassed gram negative non-fermenting organisms such as Pseudomonas spp. Burkholderia spp., and Acinetobacter spp.
5. Other
   1. Any other organisms identified from routine microbiology including fungal and mycobacterial species.
6. None
   1. Isolation of a single pathogen.

Supplementary Results

**Sup Figure 1**. Geographical boundaries of the Top End Health Service with the three major sites highlighted.


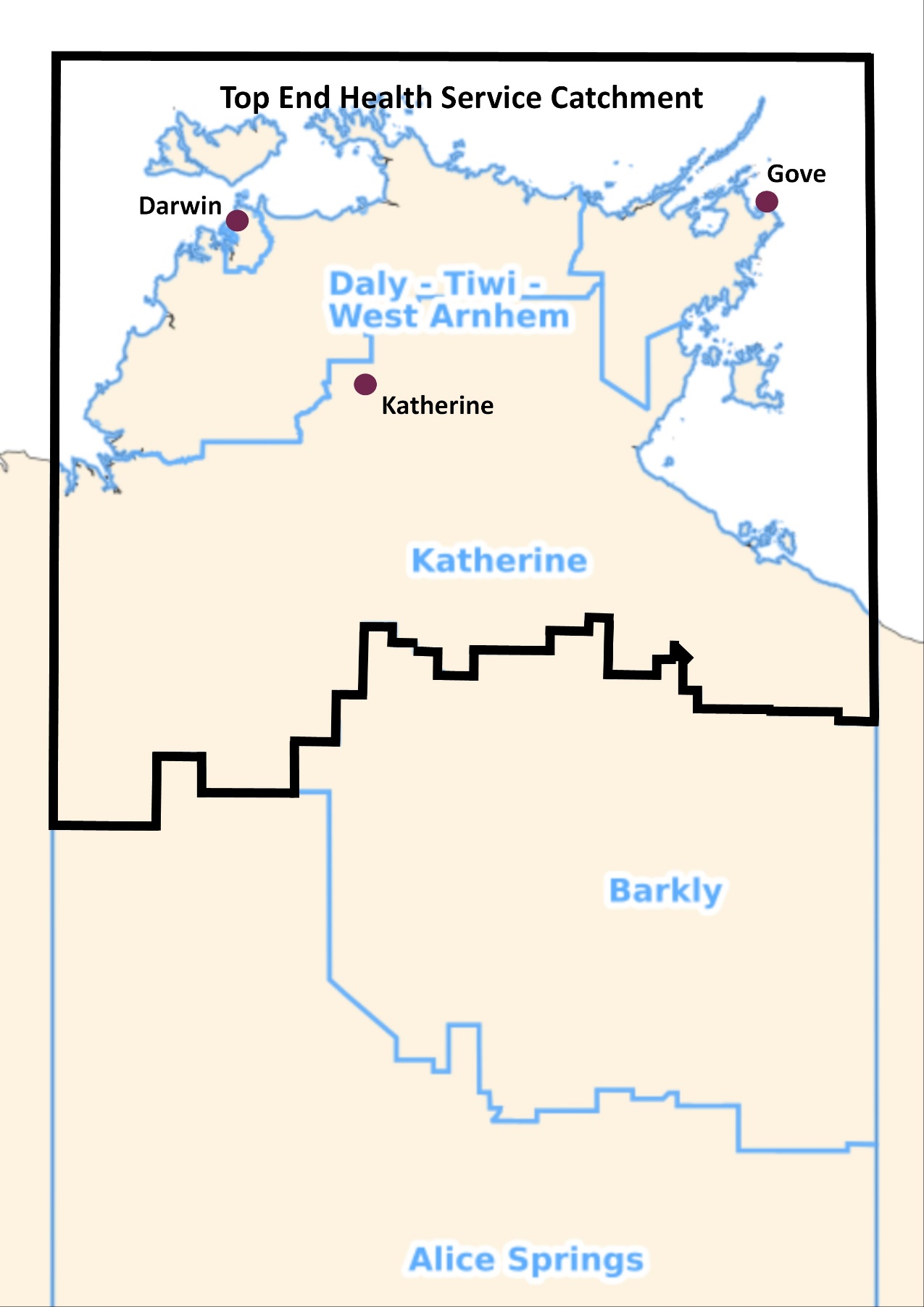


| **Sup. Table 1.** Cross tabulation of effective antimicrobial therapy and water exposure for all VACS organisms. | | | | | |
| --- | --- | --- | --- | --- | --- |
|  |  | VACS effective antibiotic given <72h | |  |  |
|  |  | No (n=167) | Yes (n=150) | Total (n=317) | p-value |
| Water Exposure | No water exposure | 117 (62.2%)* | 71 (37.7%)* | 188 | <0.001 |
|  | Salt | 15 (31.9%)* | 32 (68.1%)* | 47 |  |
|  | Fresh | 9 (27.3%)* | 24 (72.7%)* | 33 |  |
|  | Unknown exposure | 22 (68.8%) | 10 (31.2%) | 32 |  |
|  | Unknown salinity | 3 (25.0%) | 9 (75.0%) | 12 |  |
|  | Brackish | 1 (20.0%) | 4 (80.0%) | 5 |  |
| *Signify statistical significance (p<0.05) in Fisher exact post hoc analysis. | | | | | |

| **Sup. Table 2.** Cross tabulation of demographic, clinical information, and outcome date by coloniser status. | | | | | | | | | |
| --- | --- | --- | --- | --- | --- | --- | --- | --- | --- |
|  | | | | |  | Infection (n=395) | Coloniser (n=22) | Total (n=317) | p-value |
| Demographic | | | | |  |  |  |  |  |
| Sex | | | | | Female | 74 (25.1%) | 7 (31.8%) | 81 (25.6%) | 0.48 |
|  |  |  |  |  | Male | 221 (74.9%) | 15 (68.2%) | 236 (74.4%) |  |
|  | | | age (Medica, IQR) | | | 42.3 (26.1-56.1) | 40.6 (31.0 | 42.2 (26.1-55.3) | 0.89 |
|  | | | Indigenous Australian | | | 108 (36.6%) | 11 (50.0%) | 119 (37.5%) | 0.21 |
|  | | | No fixed address | | | 10 (3.4%) | 1 (4.5%) | 11 (3.5%) | 0.78 |
| Comorbidities | | | | |  |  |  |  |  |
|  | Cigarette use | | | | | 64 (21.7%) | 7 (31.8%) | 71 (22.4%) | 0.27 |
|  | Chronic Lung Disease | | | | | 21 (7.1%) | 6 (27.3%) | 27 (8.5%) | 0.001 |
|  | Heart failure | | | | | 10 (3.4%) | 3 (13.6%) | 13 (4.1%) | 0.019 |
|  | Chronic Liver Disease | | | | | 11 (3.7%) | 2 (9.1%) | 13 (4.1%) | 0.22 |
|  | Chronic Kidney Disease | | | | | 21 (7.1%) | 4 (18.2%) | 25 (7.9%) | 0.063 |
|  | Haemodialysis | | | | | 4 (1.4%) | 2 (9.1%) | 6 (1.9%) | 0.01 |
|  | Immunocompromise | | | | | 12 (4.1%) | 1 (4.5%) | 13 (4.1%) | 0.91 |
|  | Diabetes | | | | | 38 (12.9%) | 7 (31.8%) | 45 (14.2%) | 0.014 |
|  | Obesity | | | | | 15 (5.1%) | 1 (4.5%) | 16 (5.0%) | 0.91 |
|  | Alcohol excess | | | | | 58 (19.7%) | 5 (22.7%) | 63 (19.9%) | 0.73 |
|  | Previous intravenous drug use | | | | | 8 (2.7%) | 1 (4.5%) | 9 (2.8%) | 0.62 |
| Injury | | | | |  |  |  |  |  |
| Site | | | | Arm | | 14 (4.7%) | 1 (4.5%) | 15 (4.7%) | 0.011 |
|  |  |  |  | Foot | | 106 (35.9%) | 4 (18.2%) | 110 (34.7%) |  |
|  |  |  |  | Groin | | 5 (1.7%)* | 3 (13.6%)* | 8 (2.5%) |  |
|  |  |  |  | Hand | | 37 (12.5%) | 2 (9.1%) | 39 (12.3%) |  |
|  |  |  |  | Head | | 7 (2.4%) | 1 (4.5%) | 8 (2.5%) |  |
|  |  |  |  | Leg | | 96 (32.5%) | 8 (36.4%) | 104 (32.8%) |  |
|  |  |  |  | Multiple | | 4 (1.4%) | 0 (0.0%) | 4 (1.3%) |  |
|  |  |  |  | Non-wound | | 7 (2.4%) | 0 (0.0%) | 7 (2.2%) |  |
|  |  |  |  | Torso | | 15 (5.1%) | 1 (4.5%) | 16 (5.0%) |  |
|  |  |  |  | Unknown | | 4 (1.4%) | 2 (9.1%) | 6 (1.9%) |  |
| Co-Pathogens | | | | |  |  |  |  |  |
|  | | | *Staphylococcus aureus* | | | 141 (47.8%) | 11 (50.0%) | 152 (47.9%) | 0.84 |
|  | | | *Streptococcus* spp | | | 73 (24.7%) | 5 (22.7%) | 78 (24.6%) | 0.83 |
|  | | | Enteric bacteria | | | 166 (56.3%) | 14 (63.6%) | 180 (56.8%) | 0.5 |
|  | | | Pseudomonads | | | 23 (7.8%) | 2 (9.1%) | 25 (7.9%) | 0.83 |
|  | | | Other | | | 136 (46.1%) | 10 (45.5%) | 146 (46.1%) | 0.95 |
|  | | | None | | | 39 (13.2%) | 3 (13.6%) | 42 (13.2%) | 0.96 |
| Treatment / Procedures | | | | | | | | | |
|  | | Antibiotics ≤72 hours` | | | | 146 (49.5%) | 4 (18.2%) | 150 (47.3%) | 0.005 |
|  | | Antibiotics ≤7 days` | | | | 159 (53.9%) | 5 (22.7%) | 164 (51.7%) | 0.005 |
|  | | Any effective antibiotics | | | | 164 (55.6%) | 5 (22.7%) | 169 (53.3%) | 0.003 |
|  | | Any surgery | | | | 124 (42.0%) | 4 (18.2%) | 128 (40.4%) | 0.028 |
|  | | Skin grafting | | | | 22 (7.5%) | 2 (9.1%) | 24 (7.6%) | 0.78 |
|  | | Amputation | | | | 9 (3.1%) | 0 (0.0%) | 9 (2.8%) | 0.41 |
| Outcomes | | | | |  |  |  |  |  |
|  | | | | shock | | 10 (3.4%) | 2 (9.1%) | 12 (3.8%) | 0.18 |
|  | | | | ICU Admission | | 13 (4.4%) | 2 (9.1%) | 15 (4.7%) | 0.32 |
|  | | | | Readmission | | 35 (11.9%) | 6 (27.3%) | 41 (12.9%) | 0.038 |
|  | | | | Length of Stay | | 3 (0-7) | 1 (0-11) | 3 (0-7) | 0.41 |
|  | | | | Died <90 days | | 6 (2.0%) | 2 (9.1%) | 8 (2.5%) | 0.042 |
| *Signify statistical significance (p<0.05) in Fisher exact post hoc analysis. | | | | | | | | | |
